# Comparison of myopic control between orthokeratology contact lenses and defocus incorporated multiple segments spectacle lenses

**DOI:** 10.7150/ijms.93643

**Published:** 2024-05-19

**Authors:** Chia-Yi Lee, Shun-Fa Yang, Yu-Ling Chang, Jing-Yang Huang, Ie-Bin Lian, Chao-Kai Chang

**Affiliations:** 1 Institute of Medicine, Chung Shan Medical University, Taichung, Taiwan.; 2 Nobel Eye Institute, Taipei, Taiwan.; 3 Department of Ophthalmology, Jen-Ai Hospital Dali Branch, Taichung, Taiwan.; 4 Department of Medical Research, Chung Shan Medical University Hospital, Taichung, Taiwan.; 5 Department of Medical Education, Cathay General Hospital, Taipei, Taiwan.; 6 Institute of Statistical and Information Science, National Changhua University of Education, Taiwan.; 7 Department of Optometry, Da-Yeh University, Chunghua, Taiwan.

**Keywords:** orthokeratology, defocus incorporated multiple segments, spherical equivalent refraction, axial length, astigmatism

## Abstract

**Purpose:** The purpose of this study was to compare the differences in myopic control effects between orthokeratology (OK) contact lenses and defocus incorporated multiple segments (DIMS) spectacle lenses.

**Methods:** A retrospective cohort study was conducted that included patients who had received OK lens, DIMS spectacle lens or single-vision spectacle treatments. A total of 54 eyes from 27 individuals, 38 eyes from 19 individuals and 42 eyes from 21 individuals were enrolled into the OK lens, DIMS and control groups, respectively. The primary outcomes were the changes in the spherical equivalent refraction (SER) and axial length (AXL) among the groups. A repeated-measure ANCOVA was adopted to calculate the SER progression and AXL elongation of the OK lens group compared with the DIMS group.

**Results:** The difference in the SER progression was clinically non-significant in the OK lens group compared with the DIMS and control groups (P = 0.001). The total AXL elongation results were similar between the OK lens and DIMS groups, but these were lower than in the control group (P = 0.005). The repeated-measure ANCOVA revealed that the SER progression difference during the study interval was clinically non-significant in the OK lens group when compared with the DIMS group (P = 0.028). The AXL elongation results between the OK lens and DIMS populations did not illustrate a significant difference (P = 0.607). In a subgroup analysis of moderate astigmatism, better AXL control was observed in the DIMS subgroup compared with the OK lens subgroup (P = 0.016).

**Conclusions:** The OK lens demonstrated a clinically non-significant effect on the SER and AXL controls compared with the DIMS spectacle lens.

## Introduction

Myopia is a globally prevalent disease. It is characterized by blurry vision at a distance that correlates with the degree of myopia 1, 2. The annual prevalence of myopia is approximately 44 percent in the United States; this increases to 80 percent in the Asian region, which has the highest rate of myopia development 3. The main pathophysiologies of myopia development and deterioration include congenital steep corneal curvature and an acquired axial elongation 4. The incidence of myopic maculopathy, optic nerve damage and retinal detachment is significantly elevated in high-myopia individuals (defined as a sphere power greater than -5.00 diopter (D)). Thus, the prevention of myopia progression and high-myopia development is necessary 5.

Certain methods have been proposed for myopia control 1, 6. The instillation of high-concentration atropine has been demonstrated to effectively control the myopia progression rate, but presents several complications such as photophobia, ocular allergy and near-range blurry vision 2, 7. The orthokeratology contact lens (OK lens) have been used for myopia control since the early 1990s, with acceptable control effectiveness 8, 9. The myopic control efficiency of OK lens for spherical equivalent refraction (SER) progression and axial length (AXL) elongation has been demonstrated to be comparable with high-concentration atropine, but without close-range blurry vision and photophobia 10. The spectacle independence rate of an OK lens is almost 100 percent, which leads to greater daily convenience 11.

The concept of a myopia-control spectacle has been debated for decades, but no effective product has been invented 8. Defocus incorporated multiple segments (DIMS) spectacle lenses were introduced in the late 2010s. They provide adequate myopic control efficiency, with a 62% retardation in AXL elongation 12, 13. The independence of eyedrop instillation and the contact lens application of DIMS spectacle lenses ensure the safety and health of the ocular surface 14. There is limited research comparing the efficiency of OK lens and DIMS spectacle lenses on myopic control. Further investigations into the possible differences between the two methods are required because of the differences in design and application 13, 15.

The purpose of our study was to evaluate the efficiency between OK lenses and DIMS spectacle lenses on myopic control using SER and AXL parameters. We analyzed the myopic control effects of the two methods using different patient populations.

## Materials and Methods

### Ethical Declaration

All the approaches in our study adhered to the Declaration of Helsinki from 1964 and its later amendments. Our study was approved by the National Changhua University of Education (project identification code: NCUEREC-112-071). The necessity of written informed consent was waived by the National Changhua University of Education because of the retrospective design of the study.

### Participant Selection

**Figure 1:**
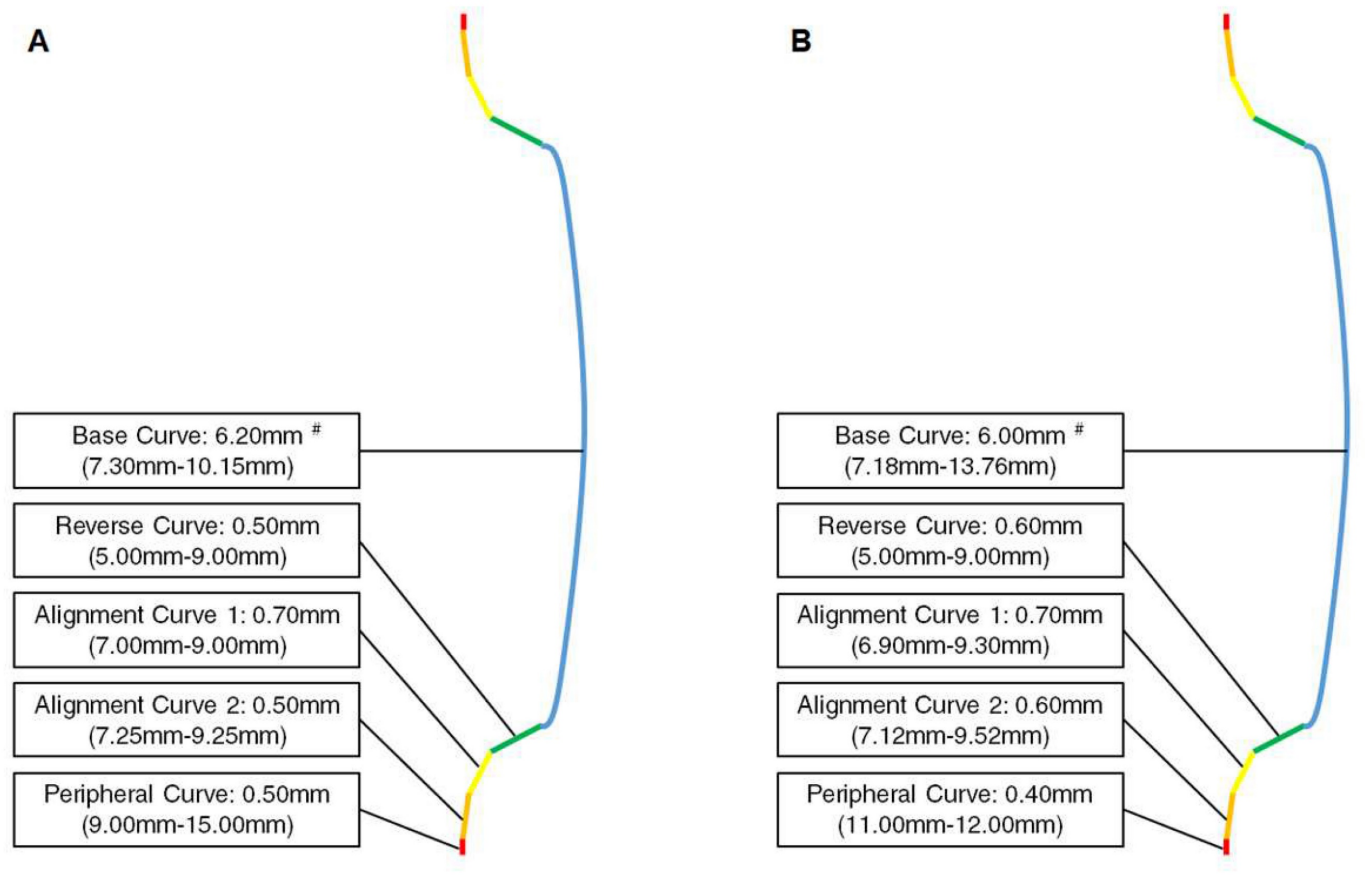
Curves of the two orthokeratology contact lenses: (A) Optical design of Euclid orthokeratology contact lens; (B) Optical design of Brighten Optix orthokeratology contact lens. ^#^ Information for each curve is presented as the optic diameter (radius of the curvature range).

**Figure 2:**
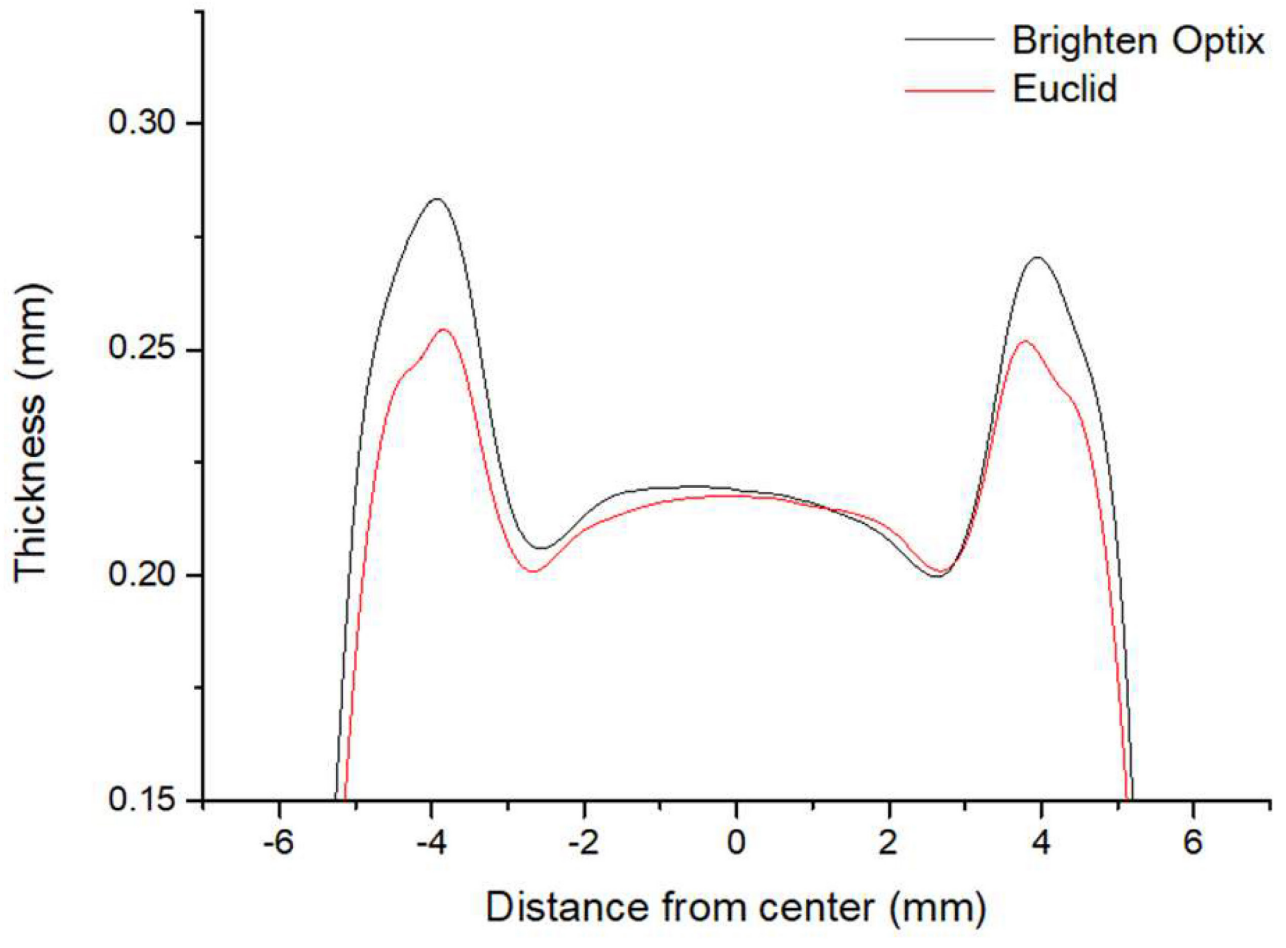
Optical designs of the two orthokeratology contact lenses.

A retrospective cohort study was performed. Patients who had received myopic control therapies at the Nobel Eye Institute were enrolled. The Nobel Eye Institute is a clinic group that has several branches in northern, central and southern Taiwan. The inclusion criteria were as follows: (1) an age younger than 15 years old, (2) the use of OK lens or DIMS spectacle lenses in our clinics and (3) regular follow-ups at our institution for at least one year. Two brands of OK lens (Euclid, Holiday Drive, Sterling, VA, US, and Brighten Optix, Shilin Dist., Taipei City, Taiwan) and one brand of DIMS spectacle lens (Miyosmart, Hoya, Shinjuku-ku, Tokyo, Japan) were used in our study. The two OK lenses consisted of five different curves, including the base curve, reverse curve, alignment curve 1, alignment curve 2 and peripheral curve. The combination of these curves, which is called the reversed geometry, is the principle used by OK lenses to control myopia 8, 9. The curves of two OK lenses—including the optic diameter and power (presented as the radius of the curvature range, which can convert to the power) of each curve—are presented in Figure 1A and Figure 1B. The optic designs of the two OK lenses were measured using a lens analyzer/deflectometry (Contest Plus II, Rotlex, Building 2D, Omer Industrial Park, Israel). The related information is presented in Figure 2. We used two brands of OK lens in our study because the efficiency of the myopic control of the two OK lenses was similar according to our clinical experience. The following exclusion criteria were adopted in our study to reduce the heterogeneity of the study population and exclude extreme conditions: (1) an initial best-corrected visual acuity (BCVA) worse than 20/25, ascertained using a Snellen chart; (2) myopia with a sphere power greater than -5.00 D; (3) astigmatism with a cylinder power greater than -2.50 D; (4) the use of atropine; and (5) severe ocular defects, including (but not limited to) corneal opacity, congenital glaucoma, juvenile uveitis, congenital cataracts, retinopathy of prematurity, persistent fetal vasculature and optic nerve atrophy. A total of 54 eyes from 27 patients and 38 eyes from 19 patients were enrolled into the OK group and DIMS group, respectively, after the selection process. For comparison, a further 42 eyes from 21 patients who wore single-vision spectacles without the use of other myopic control tools (including atropine) were selected as the control group. We included both eyes of one participant because the refraction status of both eyes was not completely identical and we wanted to include as many cases (eyes) as possible.

### Primary Outcomes

The baseline characteristics of each participant—including age, sex, pre-treatment BCVA, sphere power, cylinder power and AXL—were collected from medical records. We used the SER progression and AXL elongation as the parameters of myopic progression; these served as the primary outcomes of our study. The SER and AXL results after cycloplegia were measured at each branch using an autorefractor (KR-8900, Topcon, Itabashi-ku, Tokyo, Japan) and a biometry device (IOL Master 500, Carl Zeiss, Göschwitzer Str., Jena, Germany), respectively. The topical cycloplegic agent tropicamide (Better eye drop, Aseptic Innovative Medicine Co. Ltd., Taoyuan dist., Taoyuan, Taiwan) was instilled at least three times before refraction. The optometrists then examined the pupil. Refraction was ascertained if the pupil diameter was greater than 8 mm. The refractive power after cycloplegia included the sphere and cylinder. This was measured three times and the average value of the three measurements was entered into the dataset of our study. The SER was calculated as the sphere power plus half of the cylinder power. The measurements in the OK group were obtained at least 7 hours after the removal of the OK lens. The SER and AXL values were obtained before the treatment as well as 1, 3, 6, 9 and 12 month/s after the treatment. These values were used in the statistical analysis.

### Statistical Analysis

SPSS version 20.0 (SPSS Inc., Chicago, Illinois, USA) was used for all the statistical analyses. A descriptive analysis was used to present the baseline characteristics among the three groups. A chi-squared test and one-way ANOVA with Dunnett's T3 post hoc test were then applied for the comparison of the baseline characteristics among the three groups. In the next step, a one-way ANOVA was used for the comparison between the initial SER and AXL, the final SER and AXL and the change in SER and AXL among the three groups, along with Dunnett's T3 post hoc test. A repeated-measure ANCOVA with a Tukey HSD test was then used to analyze the trend in SER progression and AXL elongation between the two groups, with adjustments for the effects of age, sex, pre-treatment BCVA, pre-treatment SER and pre-treatment AXL. In the subgroup analysis, the SER and AXL of patients who had received an OK lens or a DIMS spectacle lens with moderate myopia (> -3.00 D), moderate astigmatism (> -1.50 D), high AXL (> 25.00 mm) and old age (> 12 years old) were separately analyzed using a generalized estimate equation. An adjusted odds ratio (aOR) with a corresponding 95% confidence interval (CI) was produced for the OK group compared with the DIMS group, with adjustments for age, sex, pre-treatment BCVA, pre-treatment SER and pre-treatment AXL. The generalized estimate equation was adopted to compare the increment in AXL (> 0.10 mm per year) of patients with moderate baseline myopia (> -3.00 D), high baseline AXL (> 25.00 mm) and old initial age (> 12 years old) with those without such conditions from the whole study population. The statistical significance level was set as P < 0.05; a P-value less than 0.001 was depicted as P < 0.001.

## Results

The baseline characteristics of the three groups are demonstrated in Table 1. The mean age was 9.87 ± 2.15 years in the OK group, 9.82 ± 2.18 years in the DIMS group and 9.90 ± 2.11 in the control group; these results did not reveal a significant difference (P = 0.792). The sex ratios among the three groups were similar (P = 0.634). The pre-treatment BCVA score was better in the DIMS and control groups compared with the OK group (P = 0.041). The other baseline characteristics were similar among the three groups (all P > 0.05) (Table 1).

**Figure 3:**
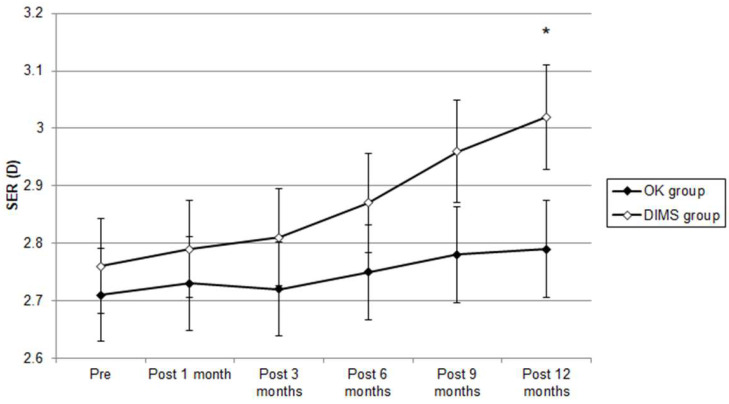
Trend of spherical equivalent refraction changes between orthokeratology contact lens and defocus incorporated multiple segments spectacle groups. D: diopter; SER: spherical equivalent refraction. * Significant difference between the two groups.

**Figure 4:**
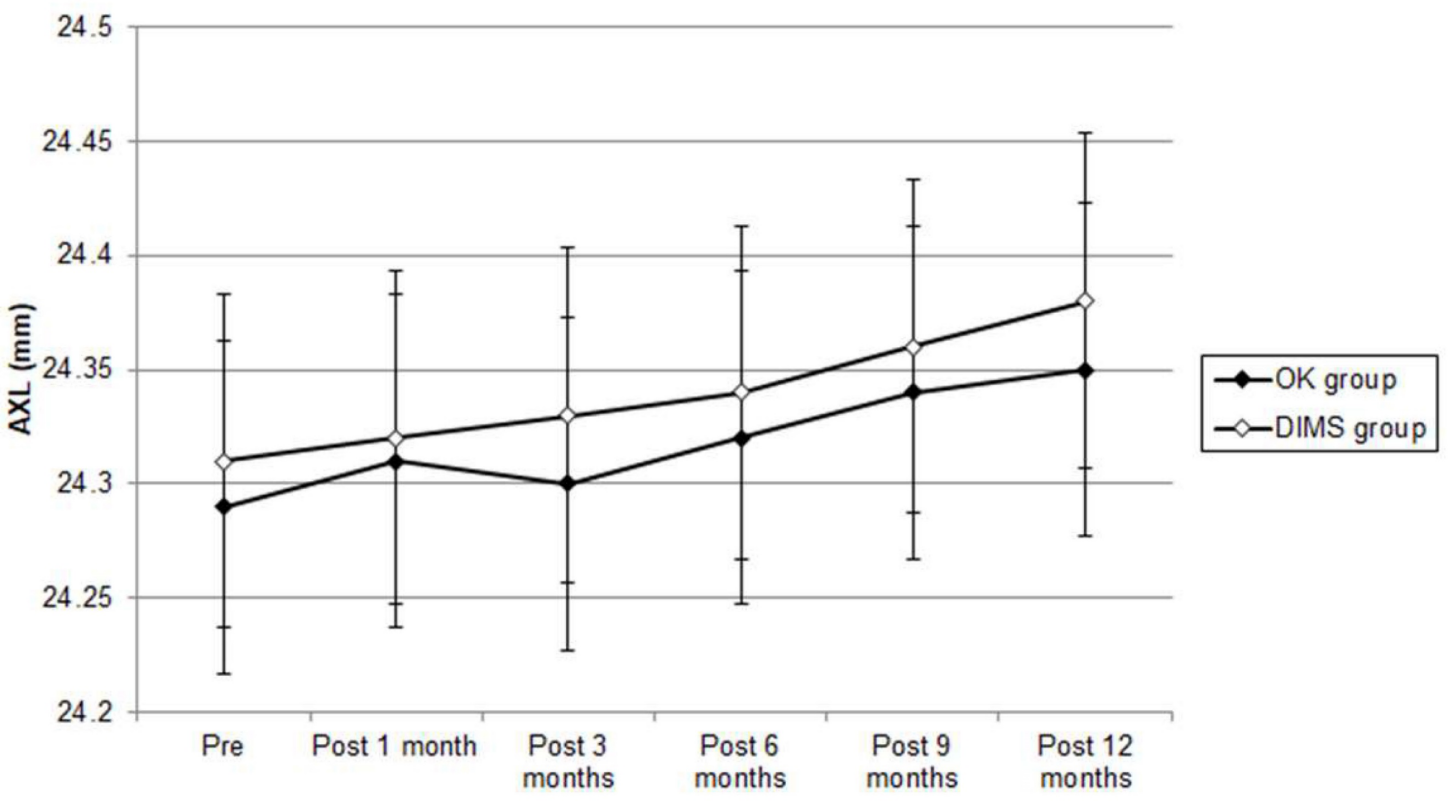
Trend of axial length change between orthokeratology contact lens and defocus incorporated multiple segments spectacle groups. AXL: axial length.

The pre-treatment SER results were -2.71 ± 1.42 D, -2.76 ± 1.39 D and -2.64 ± 1.33 D in the OK, DIMS and control groups, respectively, and did not present a significant difference (P = 0.212). After the entire follow-up period, the SER progression results were -0.08 ± 0.32 D, -0.26 ± 0.51 D and -0.57 ± 0.69 D in the OK lens, DIMS and control groups, respectively. The difference in SER progression was clinically non-significant among the three groups (P = 0.001). The pre-treatment AXL results were 24.29 ± 0.85 mm, 24.31 ± 0.82 mm and 24.27 ± 0.80 mm in the OK lens, DIMS and control groups, respectively; the three groups presented a similar initial AXL (P = 0.381). One year after starting the treatment, the AXL elongation was 0.06 ± 0.31 mm in the OK group, 0.07 ± 0.58 mm in the DIMS group and 0.16 ± 0.45 mm in the control group. The degrees of AXL elongation in the OK lens and DIMS groups were significantly lower than the control group (P = 0.005) (Table 2). The SER progression difference during the study interval was clinically non-significant in the OK group compared with the DIMS group (P = 0.028) from the repeated-measure ANCOVA that was adjusted for several confounders (Figure 3). The AXL elongation between the OK and DIMS populations did not illustrate a significant difference, according to the repeated-measure ANCOVA (P = 0.607) (Figure 4). The effect on the myopic control of the OK lens and DIMS groups for the SER and AXL (by percentages) are presented in Table 3.

In the subgroup analysis, there were 21, 9, 19 and 14 eyes in the OK group and 18, 7, 16 and 8 eyes in the DIMS group belonging to the moderate myopia, moderate astigmatism, high AXL and old age subgroups, respectively. The difference in SER progression was clinically non-significant in the OK population compared with the DIMS population in all subgroups (all P < 0.05) (Table 4). The moderate-astigmatism patients with an OK lens application presented a higher AXL elongation compared with the moderate-astigmatism participants with DIMS (aOR: 1.915; 95% CI: 1.168-2.930; P = 0.016). The AXL elongation was similar in the OK and DIMS populations in the other subgroups (all P > 0.05) (Table 4). A faster AXL elongation was associated with a younger initial age (aOR: 0.637; 95% CI: 0.258-0.894; P = 0.009) and a higher baseline SER (aOR: 1.524; 95% CI: 1.107-1.872; P = 0.005) but not a higher baseline AXL (aOR: 1.359; 95% CI: 0.883-2.004; P = 0.094).

## Discussion

The SER progression difference was clinically non-significant in the OK group in our study. The AXL elongation was similar between the two populations. Both the OK lens and DIMS spectacle lens groups demonstrated acceptable efficiency for myopic control 14, 16. The use of OK lenses demonstrated comparable SER progression and AXL elongation results with high-concentration atropine instillation and spectacle independence 17. The use of DIMS spectacle lenses has previously been demonstrated to contribute to a more than 60 percent reduction in AXL elongation after two years compared with patients who received a single-vision spectacle lens 12. Studies directly comparing the efficiency of the two methods on myopic control are rare. We excluded patients with atropine applications to remove the significant effect of myopic retardation from atropine 5. We also adjusted for several confounders—including age and initial refractive status—in the repeated-measure ANCOVA. Consequently, the similar results for the SER progression and AXL elongation between the OK and DIMS groups could be credible. Corneal curvature decreases after the application of an OK lens and persists for a period of time 18-20. The numerically lower SER amount in the OK group may have been a result of the flattening effect of the OK lens because we could not prevent the application of OK lenses for the SER measurements on every visit. Both the OK and DIMS groups presented fair control effects, with the AXL elongation lower than 0.1 mm within one year. AXL is the most accurate parameter for the progression of acquired myopia 21. The similar AXL control effect of the two groups may further indicate the same efficiency in the myopia control of the two methods.

The SER progression rates were clinically non-significant in all the subgroup analyses when comparing OK lens applications with DIMS spectacle lens use. This may have been a result of the cornea-flattening effect of the OK lens, as described previously 18. Similarly, the difference in the AXL elongation between the OK lenses and DIMS spectacle lenses in each subgroup did not reveal a significant difference. The exception was the DIMS spectacle lens, which demonstrated a higher AXL control efficiency in patients with moderate astigmatism, with a cylinder power greater than -1.50 D. One other publication reported similar results with an adequate follow-up frequency of six times in one year. Individuals with moderate astigmatism usually require a toric OK lens to correct and control myopia 22. The efficiency of toric OK lenses for myopic control has been demonstrated to be similar to that for non-toric OK lenses 23. A toric OK lens could correlate with the development of higher-order aberrations 24. A reduced visual quality is a risk factor for myopic progression; thus, the efficiency of a toric OK lens on AXL retardation might be influenced 8. The DIMS spectacle lens does not cause higher-order aberrations and there has been no prior report of visual disturbances caused by DIMS spectacle lenses 25. It is possible that the absence of higher-order aberrations and the visual comfort degree in children who received a DIMS spectacle lens treatment could be associated with better patient compliance. Accordingly, the effect of myopic control for AXL elongation is likely better for DIMS than OK lens patients with moderate astigmatism. No moderate-astigmatism patients who received an OK lens treatment developed OK-lens-related keratitis, including during the follow-up period 26, 27. This implies that there is an acceptable safety level for OK lens treatments on individuals with moderate astigmatism.

The rates of SER progression were -0.08 ± 0.32 D and -0.26 ± 0.51 D in the OK and DIMS groups in our study, respectively, for the overall efficiency of myopic control. Previous research demonstrated an annual SER progression of -0.22 ± 0.54 D in patients who received high-concentration atropine therapy, whereas another study presented an annual SER progression of +1.01 ± 0.87 D in patients who received OK lens treatments 5, 28. The rates of SER progression in both the OK and DIMS groups in our study were compatible with previous research 5, 28. Although the degree of SER progression was numerically higher in the DIMS group than in previous results 5, 28, the difference (lower than -0.50 D) may not cause a significant deterioration in visual acuity and refractive status in clinical practice. The changes in AXL have ranged from 0.02 mm to 0.27 mm annually in previous studies using high-concentration atropine and OK lenses 2, 5. The AXL elongation results of the OK and DIMS groups in our study were 0.06 ± 0.31 mm and 0.07 ± 0.58 mm, respectively; these were comparable with previous results 2, 5. Previous research combined low-concentration atropine and OK lenses, resulting in SER progression and AXL elongation outcomes of 0.88 ± 0.31 D and 0.50 ± 0.17 mm, respectively, in two years 29. Our SER progression and AXL elongation results were not inferior to those in that study 29, which could indicate that a single method for myopia control with either OK lenses or DIMS spectacle lenses is adequate for the general myopic population.

The pre-treatment BCVA was significantly better in the DIMS group compared with the OK lens group in our study. Although a statistical significance was reached in the DIMS group, the difference was only 0.01 LogMAR and did not present any clinical significance. Myopia is a growing disease, especially in the eastern Asian region 3. The rate of myopic progression in individuals younger than 7 years of age is significantly faster than in children aged 10-11 years; this is similar to our analysis comparing AXL elongation and an older initial age 30. A DIMS spectacle lens may serve as a prevention and treatment method in young children with early myopia development rather than low-concentration atropine because it can be applied to younger individuals compared with the OK lens.

There were a few limitations to our study. First, the retrospective design may have caused a higher heterogeneity among the study population compared with a prospective study. Second, the pupillary diameter was not measured in our study because of the retrospective nature (we did not perform this examination in our routine work). This may have reduced the integrity of our results. The absence of a retinoscopy exam after cycloplegia could reduce the accuracy of refraction. The patients in our study were recruited from different branches and managed by different ophthalmologists; thus, the judgment and management methods used may have been different. Finally, the influence of different ages might not be prominent in our analysis because the age among the OK, DIMS and control groups presented only a small difference without statistical significance, and we adjusted for the effect of age in the repeated-measure ANCOVA and generalized estimate equation. Thus, the influence of different age in our analysis might not be prominent.

In conclusion, the application of an OK lens contributed to a clinically non-significant difference in both SER and AXL controls compared with the use of a DIMS spectacle lens. DIMS spectacle lenses demonstrated a better control of AXL elongation compared with OK lenses in individuals with moderate astigmatism. Consequently, the choice of an OK lens or a DIMS spectacle lens for myopic control could be based on the condition of each patient. A further large-scale prospective study is mandatory to evaluate whether OK lenses and DIMS spectacle lenses present different myopic control effects in individuals with high myopia.

## Figures and Tables

**Table 1:** Basic characteristics among the three groups.

Characteristic	OK Group (N = 54)	DIMS Group (N = 38)	Control Group (N = 42)	P-Value
Age	9.87 ± 2.15	9.82 ± 2.18	9.90 ± 2.11	0.792
Sex (male/female)	10:17	6:13	8:13	0.634
Pre-treatment BCVA (LogMAR)	0.01 ± 0.04	0.00 ± 0.01^a^	0.00 ± 0.03^a^	0.041*
Pre-treatment sphere (D)	-2.33 ± 1.39	-2.36 ± 1.43	-2.28 ± 1.24	0.338
Pre-treatment cylinder (D)	-0.76 ± 0.77	-0.79 ± 0.74	-0.72 ± 0.75	0.465
Hours of device use	8.12 ± 0.49	8.38 ± 0.70	8.40 ± 0.32	0.277

AXL: axial length; D: diopter; DIMS: defocus incorporated multiple segments; N: number; OK lens: orthokeratology contact lens. * Significant difference among groups. ^a^ Similar statistical values.

**Table 2:** Changes in spherical equivalent refraction and axial length among the three groups after the follow-up period.

Outcome	OK Group (N = 54)	DIMS Group (N = 38)	Control Group (N = 42)	P-Value
SER (D)				
Pre-treatment	-2.71 ± 1.42	-2.76 ± 1.39	-2.64 ± 1.33	0.212
Post-treatment	-2.79 ± 1.44	-3.02 ± 1.46	-3.21 ± 1.42	0.002*
Change	-0.08 ± 0.32	-0.26 ± 0.51	-0.57 ± 0.69	0.001*
AXL (mm)				
Pre-treatment	24.29 ± 0.85	24.31 ± 0.82	24.27 ± 0.80	0.381
Post-treatment	24.35 ± 0.73^a^	24.38 ± 0.91^a^	24.43 ± 0.88	0.019*
Change	0.06 ± 0.31^a^	0.07 ± 0.58^a^	0.16 ± 0.45	0.005*

AXL: axial length; D: diopter; DIMS: defocus incorporated multiple segments; N: number; OK lens: orthokeratology contact lens; SER: spherical equivalent refraction. * Significant difference among groups. ^a^ Similar statistical values.

**Table 3:** Percentage of spherical equivalent refraction and axial length elongation reduction in OK lens and DIMS groups.

Tool	SER Reduction (%)	AXL Reduction (%)
OK lens	85.97	54.39
DIMS	62.50	56.25

AXL: axial length; DIMS: defocus incorporated multiple segments; OK lens: orthokeratology contact lens; SER: spherical equivalent refraction.

**Table 4:** Subgroup analysis between OK lens and DIMS groups with different characteristics.

Subgroup	Difference^†^	aOR^#^	95% CI	P-Value
SER (D)				
Moderate myopia	0.21	0.325	0.120-0.683	0.001*
Moderate astigmatism	0.15	0.664	0.432-0.917	0.017*
High AXL	0.16	0.578	0.104-0.828	0.004*
Old age	0.19	0.435	0.286-0.521	0.002*
AXL (mm)				
Moderate myopia	0.01	1.396	0.227-2.329	0.491
Moderate astigmatism	0.03	1.915	1.168-2.930	0.016*
High AXL	0.01	1.562	0.433-2.275	0.383
Old age	< 0.01	1.227	0.145-1.758	0.562

aOR: adjusted odds ratio; AXL: axial length; CI: confidence interval; D: diopter; SER: spherical equivalent refraction. * Significant difference between groups. ^†^ Mean change in the OK subgroup minus the mean change in the DIMS group. ^#^ OK group versus DIMS group, adjusted for age, sex, pre-treatment BCVA, pre-treatment SER and pre-treatment AXL.

## Abbreviations

D: diopter
OK: orthokeratology
SER: spherical equivalent refraction
AXL: axial length
DIMS: defocus incorporated multiple segments
aOR: adjusted odds ratio
CI: confidence interval
N: number
